# The Science of Shiga Toxin-Producing (Verotoxin-Producing) *Escherichia coli* (STEC): An Ongoing One Health Journey toward Improved Health and Food Safety—Editorial Summary

**DOI:** 10.3390/microorganisms12020344

**Published:** 2024-02-07

**Authors:** Krysty D. Thomas, Tim A. McAllister

**Affiliations:** Agriculture and Agri-Food Canada, Lethbridge Research and Development Centre 5403 1st Ave South, Lethbridge, AB T1J 4B1, Canada; krysty.thomas@agr.gc

Verotoxigenic *Escherichia coli* (VTEC), also termed Shiga toxin-producing *Escherichia coli* (STEC), is a human pathogen transmitted by food, water, animals, and their environment, and from one person to another [1]. The pathogen typically causes diarrheal illness but can also cause severe systemic disease, particularly in children and the elderly [1]. Virulence is associated with a type III secretion system, which enables injection of bacterial effector proteins into host cells [2]. In addition, Shiga toxins can damage the kidneys and lead to hemolytic uremic syndrome (HUS). No specific treatment is available for STEC infection. To date, there have been advances in the epidemiology, pathogenesis, and genomics of STEC, many of which are discussed in this Special Issue: “The Science of Shiga Toxin-Producing (Verotoxin-Producing) Escherichia coli (STEC): An Ongoing One Health Journey toward Improved Health and Food Safety”, with new data and novel methodologies and technologies to enhance our understanding. In the present day, dangerous outbreaks still occur. In September 2023, Canada experienced an outbreak linked to children daycares which resulted in 446 cases of the disease, with 38 children hospitalized and 23 diagnosed with HUS; fortunately, there were no fatalities [3].

In order to reduce the incident of human illness, a better understanding of the pathogenesis and epidemiology of STEC infection is needed, with an emphasis on a One Health-approach solution to the disease. This Special Issue of *Microorganisms* gathers six articles addressing various aspects of STEC research, related to its pathogenesis and epidemiology (1–3), metagenomics (4), host interactions (4), and the persistence of STEC in the environment (5). Much of this work answers critical questions related to the complex interactions between pathogen, humans, animals, and the environment.

Future STEC research should focus on (i) strengthening integrated surveillance systems that encompass human, animal, and environmental monitoring to better understand STEC epidemiology; (ii) investigating host immune responses to different STEC strains to identify potential targets for therapeutic interventions; (iii) continued research on reservoirs and transmission dynamics; (iv) studying antimicrobial resistance patterns in STEC strains and understanding their implications for treatment and control measures, (v) investing in research for the development of vaccines against STEC to prevent infections or reduce the severity of disease; (vi) fostering international collaboration to share data, resources, and expertise for a more comprehensive global understanding of STEC . Scientific meetings, for example, the International Symposium on Shiga Toxin (Verocytoxin) Producing *E. coli* Infections (VTEC 2023) held in May 2023 in Banff, Alberta, Canada, should continue. This meeting is linked to the current Special Issue and enabled over 200 researchers from around the world to share data, collaborate, and develop solutions regarding STEC. The next triennial conference is scheduled in Aberdeen, the UK, in 2026. 
